# Analysis of the Host Transcriptome from Demyelinating Spinal Cord of Murine Coronavirus-Infected Mice

**DOI:** 10.1371/journal.pone.0075346

**Published:** 2013-09-18

**Authors:** Ruth Elliott, Fan Li, Isabelle Dragomir, Ming Ming W. Chua, Brian D. Gregory, Susan R. Weiss

**Affiliations:** 1 Department of Microbiology, Perelman School of Medicine, University of Pennsylvania, Philadelphia, Pennsylvania, United States of America; 2 Department of Biology, University of Pennsylvania, Philadelphia, Pennsylvania, United States of America; National Institutes of Health, United States of America

## Abstract

Persistent infection of the mouse central nervous system (CNS) with mouse hepatitis virus (MHV) induces a demyelinating disease pathologically similar to multiple sclerosis and is therefore used as a model system. There is little information regarding the host factors that correlate with and contribute to MHV-induced demyelination. Here, we detail the genes and pathways associated with MHV-induced demyelinating disease in the spinal cord. High-throughput sequencing of the host transcriptome revealed that demyelination is accompanied by numerous transcriptional changes indicative of immune infiltration as well as changes in the cytokine milieu and lipid metabolism. We found evidence that a Th1-biased cytokine/chemokine response and eicosanoid-derived inflammation accompany persistent MHV infection and that antigen presentation is ongoing. Interestingly, increased expression of genes involved in lipid transport, processing, and catabolism, including some with known roles in neurodegenerative diseases, coincided with demyelination. Lastly, expression of several genes involved in osteoclast or bone-resident macrophage function, most notably TREM2 and DAP12, was upregulated in persistently infected mouse spinal cord. This study highlights the complexity of the host antiviral response, which accompany MHV-induced demyelination, and further supports previous findings that MHV-induced demyelination is immune-mediated. Interestingly, these data suggest a parallel between bone reabsorption by osteoclasts and myelin debris clearance by microglia in the bone and the CNS, respectively. To our knowledge, this is the first report of using an RNA-seq approach to study the host CNS response to persistent viral infection.

## Introduction

Mouse hepatitis virus (MHV) is a positive-sense RNA virus of the coronaviridae family. MHV strain A59 is both hepatotropic and neurotropic, and intracranial inoculation of mice causes acute hepatitis and encephalitis and subsequent demyelination. MHV-,A59 replicates in the brain, spinal cord and liver, peaking at about five days post infection after which time infectious virus is cleared primarily by the CD8T cell response, also requiring CD4T cells. Although virus is cleared by 10-14 dpi from all organs, viral RNA persists in the central nervous system (CNS) and is accompanied by inflammatory demyelination, which peaks in the spinal cord at approximately one month post-infection [1-6]. Demyelinating strains of MHV (A59 and JHM) have been widely used as models for the study of the human demyelinating disease multiple sclerosis [1,7-10]. MHV-induced CNS disease involves recurring demyelination and remyelination in spinal cords of infected mice [5,6,11], suggesting that this model has pathological similarities to the remitting-relapsing forms of multiple sclerosis.

The mechanisms underlying MHV-induced demyelination remain unclear. Although MHV induces primary demyelination, axonal damage has also been observed in MHV-infected CNS [12]. Early pathological studies suggested that demyelination results from direct infection of and damage to oligodendrocytes, the myelin producing cells of the CNS [13,14]. More recent reports have implicated immunopathological mechanisms rather than oligodendrocyte damage in MHV-induced demyelination, although the specific pathways involved still remain to be determined. While RAG-/- mice, lacking mature B- and T-cells, do not undergo demyelination, neither CD4 nor CD8T-cells are absolutely required [2,8]. Virus-specific T cells persist in the CNS [15,16], and both macrophages and microglia are associated with demyelinating lesions [14]. Mice lacking B-cells exhibit more severe demyelination than immunocompetent mice, throughout the course of infection [2], but this is likely due at least in part to persistence of infectious virus. Additionally, several cytokines have been studied in the context of MHV-induced demyelination; only IP-10 is reported to affect the clinical course of disease [17-21]. While many demyelinating disorders are also presumed to involve dysregulation of lipid metabolism and transport [22-25], there are to date no reports of alterations of lipid metabolism during MHV infection.

To comprehensively characterize the gene expression changes and pathways affected by MHV-induced demyelination in the murine CNS, we employed high-throughput RNA sequencing (RNA-seq) using spinal cords from MHV-A59-infected mice at 33 days post-infection, around the time of maximum MHV-induced demyelination as quantified luxol fast blue staining of spinal cord sections [1,2,5,26]. At this time post infection, demyelination is robust and viral genome expression is detectable [1,2,5,16,26]. To our knowledge, this is the first report of using RNA-seq to detail the host response to virus-induced demyelinating disease. We found that the host response to MHV persistence was characterized by a Th1-biased cytokine response, eicosanoid-associated inflammation, ongoing antigen presentation, lymphocyte proliferation and activation, and lipid processing changes. In addition, the expression levels of several genes involved in osteoclast function, or the function of bone-resident macrophages, were augmented in the demyelinating CNS, most likely being expressed by brain resident microglia. Two of these genes, TREM2 and DAP12, were among the most highly induced during MHV-induced demyelinating disease. Interestingly, natural polymorphisms in these genes lead to a demyelinating disorder in humans concurrent with osteopetrosis, or bone cysts [27-30]. The increased expression of genes that directly affect bone resorption and osteoclasts during demyelination leads to the intriguing possibility that osteoclasts and microglia may have parallel roles in maintaining the specialized extracellular matrix of their respective compartments.

## Materials and Methods

### Mice, viruses, and cells

C57/Bl6 (B6) mice were obtained from the National Cancer Institute (Frederick, MD). Four-week-old mice were inoculated intracranially with 2000 plaque-forming units (pfu) of MHV-A59 [7] or an equal volume of uninfected cell lysate (mock infected) in 25 µL of PBS containing 0.75% BSA, and tissues were harvested at the indicated dpi. Tissues were flash frozen for later RNA isolation, fixed in phosphate buffered formalin for tissue embedding, or frozen in saline with 0.167% gelatin for titering of infectious virus. Standard plaque assays were performed on transformed mouse fibroblast L2 cells as described previously [31].

### Ethics Statement

All mouse procedures were performed on protocols approved by the University of Pennsylvania’s Institutional Animal Care and Use Committee (IACUC) (Reference Assurance # A3079-01). Intracranial inoculations were carried out under anesthesia with isoflurane and all efforts were made to minimize suffering. The University Of Pennsylvania School Of Medicine is fully accredited by the Association for the Assessment and Accreditation of Laboratory Animal Care International (AAALAC).

### Isolation of primary murine cells

Bone marrow-derived macrophages (BMM) were generated from the hind limbs of B6 mice as described previously [32,33]. Briefly, bone marrow was differentiated into macrophages by growth in L929 fibroblast secreted M-CSF. Macrophages were seeded after 6-7 days *in* *vitro*.

Neurons were isolated from the hippocampi of E15.5-16.5 mouse embryos and plated on poly-L-lysine coated wells without astrocyte feeder layers as described previously [34,35]. Hippocampal neuron cultures were typically >95% pure as assessed by immunostaining with an antibody recognizing MAP2 (1:2; AP14, kindly provided by Virginia Lee, University of Pennsylvania).

Primary glial cultures were established as described previously [36], with the following modifications. For isolation of astrocytes, confluent mixed glial cultures were shaken on day 4-7 *in vitro* to dissociate the loose top layer of microglia, which were discarded. The remaining adherent cells, mostly astrocytes, were trypsinized and re-plated at a 1:2 dilution. This procedure was repeated on day 13-15. Prior to seeding, the astrocytes were passed through a series of three suspension cell culture plates for 20 minutes each at room temperature to allow adhesion of contaminating microglia and fibroblasts. For isolation of microglia, mixed glial cultures were shaken on d13-15 and the cells in the supernatant re-plated. The medium was replaced after 30 minutes to remove contaminating astrocytes, which adhere more slowly. Astrocyte and microglial cultures were each >95-99% pure as assessed by immunofluorescence staining with monoclonal antibodies directed against GFAP for astrocytes (Dako Z0334) and CD11b for microglia (Abcam ab6332) as described previously [35].

Mouse oligodendrocyte cultures were derived from a mixed population of cells isolated from the forebrains of 1- to 3-day-old neonatal C57/BL6 mice and cultured in serum-free growth medium containing PDGF, FGF2, and NT-3, as previously described [37]. When confluent, the oligodendrocyte-lineage cells were purified using a modified wash down procedure to remove astrocytes and other contaminating cells [37].

### Persistence experiment

Brain lysates were cleared of cell debris by centrifugation at 3,000 rpm for fifteen minutes. A portion of the supernatant was removed and centrifuged at 13,000 rpm for three minutes. Twenty microliters of this supernatant was then injected into each of 3-5 naïve four-week old B6 mice. These mice were observed for clinical symptoms of disease, and their brains, spinal cords, and livers were harvested at the indicated dpi. Extracts from the second round of mice were titered by standard plaque assay and assayed for characteristic cytopathic effects of MHV on L2 mouse fibroblast cells. The L2 fibroblast lysates were then assayed for viral RNA using polymerase chain reaction (PCR) with primers specific to the viral subgenomic mRNA7.

### Real time quantitative reverse transcriptase-PCR (qRT-PCR)

Total RNA was isolated from homogenized tissue or cultured cells using either RNeasy mini kit columns (Qiagen) or TRIzol (Gibco) according to the manufacturer’s instructions and depleted of DNA using Turbo DNase (Ambion). qRT-PCR was performed as described previously [38] on each biological replicate. Briefly, 350 ng of total RNA (from cells or tissue) was transcribed into cDNA using the Superscript III reverse transcript kit (Invitrogen). Then, 2 µl of cDNA was combined with 12.5 µl of iQ5 SYBR green mix (Bio-Rad, Hercules, CA), 6.5 µl of DEPC-treated water, and 4 µl of mixed primers (5 µM each), DNA was amplified using an iQ5 iCycler (Bio-Rad), and cycle threshold (*C*_*T*_) values were recorded. Basal mRNA levels were expressed as Δ*C*_*T*_, that is, relative to the level of β-actin mRNA (Δ*C*_*T*_= *C*_*T*（gene of interest）_-*C*_*T*（ß-actin）_). The expression levels in infected spinal cords relative to those in mock-infected spinal cords (fold changes) were expressed as 2^-∆∆CT^.

### Illumina library preparation and sequencing

Tissues were solubilized in TRIzol, and total RNA was isolated and DNase-treated as described above. Poly-A-containing mRNA was selected from 20 µg of RNA pooled from 5 mice using the Dynabeads mRNA direct kit (Invitrogen). The mRNA was fragmented, a 5’ phosphate was added using T4 polynucleotide kinase (Invitrogen), and the library was size-selected (60-200 nucleotides) on a 15% TBE-urea gel. Illumina v1.5 RNA adapters were ligated to ends of the fragmented products using T4 RNA ligase 2 (New England Biolabs). A cDNA library was generated using Superscript II (Invitrogen) and PCR-amplified using Phusion HiFi DNA polymerase (New England Biolabs). The library was purified on a 6% TBE gel (Invitrogen). The quality of the library was verified prior to Illumina sequencing by cloning into Zero Blunt TOPO (Invitrogen) and analyzing 6-10 clones by standard sequencing to determine a) the presence of adapters, b) appropriate size selection, c) the abundance of ribosomal RNA, and d) the diversity of the library products. The verified size-selected poly-A stranded cDNA library was submitted to the Penn Genome Frontiers Institute (PGFI, University of Pennsylvania, Philadelphia, PA) for sequencing. The Illumina Solexa GAII platform was used to obtain 50-bp single-end sequencing reads, which were analyzed using the v1.5 pipeline.

### Computational Analysis

The reads were aligned to the mm9 genome using Bow tie [39]. Differential expression was analyzed using DESeq [40] in the R statistical environment (www.r-project.org/). Transcripts with a fold induction ≥ 2 and Benjamini-Hochbery adjusted p-value ≤ 0.05 were considered significant and included in downstream analysis. Gene ontology (GO) analysis [41] was performed using the Database for Annotation, Visualization, and Integrated Discovery (DAVID) online resource. Pathway analysis was also performed using DAVID [42], and enriched categories and pathways were further examined by qRT-PCR analysis. Genes involved in myelination, osteoclast function, oligodendrocyte differentiation, lipid/cholesterol transport and metabolism, and cytokine activity were queried from the GO database (http://www.geneontology.org/), and the resulting *Mus musculus* gene information from these lists were used to assess the expression of these genes in the MHV datasets. The data were deposited in the Gene Expression Omnibus (GEO) at the National Center for Biotechnology under accession number GSE44333.

## Results

### MHV genome and mRNA, but not infectious virus, persist in the central nervous system (CNS) of infected B6 mice

MHV persistence is a well-documented phenomenon, but the extent to which viral transcription and translation occur at late time points (one month or later) post infection are not clear [1,13,43]. Here, we have employed a highly sensitive assay for detection of infectious virus in conjunction with targeted amplification of genomic and messenger RNA to investigate the presence of viral components in the spinal cord during chronic disease. Four-week-old C57Bl/6 (B6) mice were inoculated intracranially with MHV-A59 and infectious viral titers in several organs quantified. During acute infection, that is, the first week, mice exhibited characteristic hunching, ataxia, depression, ruffled fur, and weight loss.

**Figure 1:**
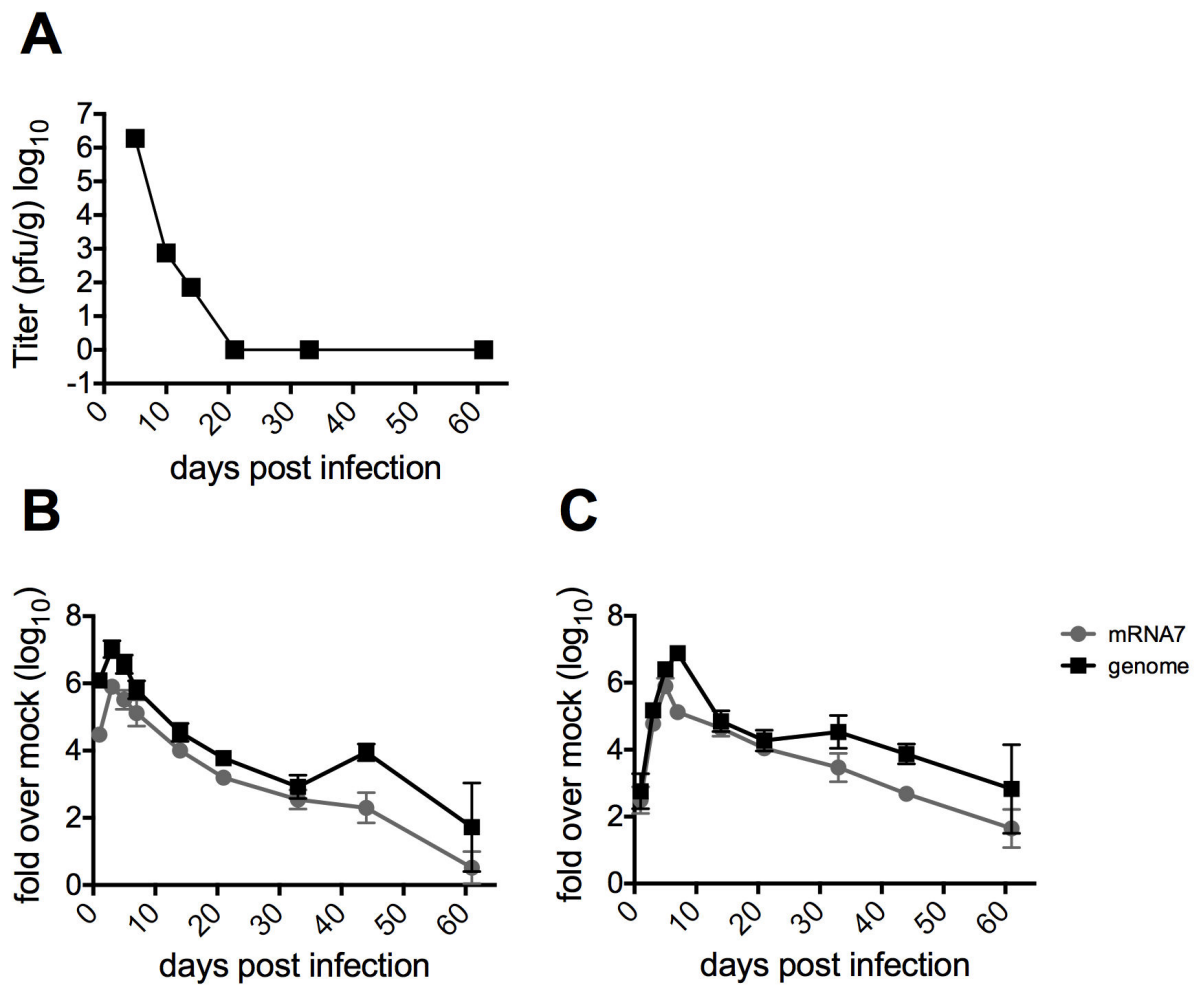
Replication of MHV in mice. Four-week old B6 mice were infected intracranially with 2000 pfu of MHV-A59 and sacrificed at the indicated days post infection. A) Brains were harvested and placed in gelatin saline, homogenized and infectious virus titered on mouse L2 fibroblasts. Titers shown are averages from five mice per group. B) Brains and C) spinal cords were harvested and RNA isolated on Qiagen RNeasy columns. qRT-PCR was performed to quantify relative abundance of MHV-A59 genomic and subgenomic RNA mRNA7. Data are plotted as means with SEM of 5-8 mice.

MHV titers peaked 5 days post infection (dpi), and by 10-14 dpi infectious virus was no longer detectable in any of the organs as assayed by standard plaque assay (Figure **1A**, data not shown). By 10-14 dpi, the mice began regaining weight and resumed their normal grooming habits and activity levels. Although infectious virus and clinical signs were no longer detectable, viral genomic RNA and subgenomic mRNAs persisted in the brain (Figure **1B**) and, to higher levels, in the spinal cord (Figure **1C**). These viral mRNAs were detectable by reverse transcriptase-quantitative polymerase chain reaction (qRT-PCR) until at least 60 dpi, suggesting a low level of ongoing transcription. During the persistent phase of infection, some mice exhibited minor weight loss, ruffled fur and slight depression, while a few mice also experienced hindlimb paresis of several days’ duration. By 60 dpi, all mice had completely recovered to similar weights as uninfected animals and exhibited normal hindlimb movement. Luxol fast blue staining of spinal cord sections taken from mice at 33 dpi, approximately at the peak of demyelinating disease [1,2,5,26] showed characteristic demyelinating lesions (data not shown).

To investigate whether very low levels of infectious virus (below the level of detection by plaque assay) are produced during the demyelinating phase of MHV infection, we used an *in vivo* bioassay to probe for infectious MHV in persistently infected tissues. Brain lysates from the above infected (donor) mice (taken on 5, 7, 14, 33, and 44 dpi) were injected into naïve four-week-old recipient mice, which were observed for clinical signs of infection (Table **1**) and sacrificed 5 and 7 dpi, at the peak times of replication and encephalitis, respectively. The recipient mice that were inoculated with brain lysates of donor mice sacrificed 5 or 14 dpi, but not from donor mice sacrificed 33 or 44 dpi, exhibited clinical symptoms of MHV disease. Recipient mice that were inoculated with 5 dpi donor mouse extract experienced weight loss, ataxia, hunching, and ruffled fur, as well as visible necrosis in the liver on dpi 7. Mice inoculated with 14 dpi donor mouse extract experienced minimal weight loss and clinical signs but had detectable titers of infectious virus in both the brain and liver 5 and 7 dpi (Table **1** and data not shown).

**Table 1:** Virus persistence in mice.

Day of isolation from donor mouse (brain)*	Day of isolation from recipient mouse (brain)**	Clinical symptoms***	Cytopathic effect of brain extract on L2 cells (recipient mouse)****
5	5	Weight loss, ataxia, hunching, ruffled fur	Yes
	7	Weight loss, ataxia, hunching, ruffled fur, necrotic liver	Yes
14	5	None, brain titer	Yes
	7	Weight loss, brain titers	Yes
33	5	No	No
	7	No	No
44	5	No	No
	7	No	No

* Four-week old mice were infected intracranially with 2000pfu of MHV-A59 and brains were harvested from donor mice on indicated day post infection.

** The cleared brain lysate from donor mice was injected into naïve four-week old recipient mice and brains were harvested from these mice on days 5 and 7 post infection.

*** Recipient mice were observed for clinical symptoms until they were sacrificed. The titers, if there were assayed, are indicated here.

**** Lysate from recipient mice was placed on L2 cells and cells were watched for cytopathic effects which include syncytia, cell death, and rounding.

Finally, the brains and livers harvested from recipient mice were assessed for infectious virus by treatment of L2 fibroblasts, which are sensitive to MHV infection, with these extracts. Cells were then assayed for viral nucleocapsid mRNA7 and genome by qRT-PCR as well as observed for cytopathic effects (Table **1**). Again, only the extracts from recipient mice that had been inoculated with extract from 5 or 14 dpi donor mice produced any cytopathic effect or viral RNA in L2 cells. These data confirm that infectious virus is not present in the CNS, as it was not detectable even by a very sensitive bioassay, during MHV-induced demyelinating disease, while both genomic and subgenomic viral RNAs remained detectable.

### MHV-induced demyelinating disease alters the transcriptome of host spinal cord tissue

To characterize the host response during the demyelinating phase of disease, total RNA was extracted from spinal cords of both mock- and MHV-infected B6 mice sacrificed 33 dpi. Poly-adenylated RNA was selected and the resulting library subjected to 50-bp Illumina Solexa GAII sequencing (Table **S1**). We used the DESeq package (http://www-huber.embl.de/users/anders/DESeq/) to analyze the changes in the host transcriptome during the demyelinating phase of MHV-A59 infection and found 909 transcripts for which expression was significantly altered (894 upregulated, 14 downregulated). Although 24,321 of the 27,731 annotated transcripts in the mouse genome (NCBI Build 37) were expressed at similar levels in mock-infected and MHV-infected animals (Figure **S1**), only 2,502 transcripts were not expressed at all in either mock-infected or MHV-infected spinal cord.

**Figure 2:**
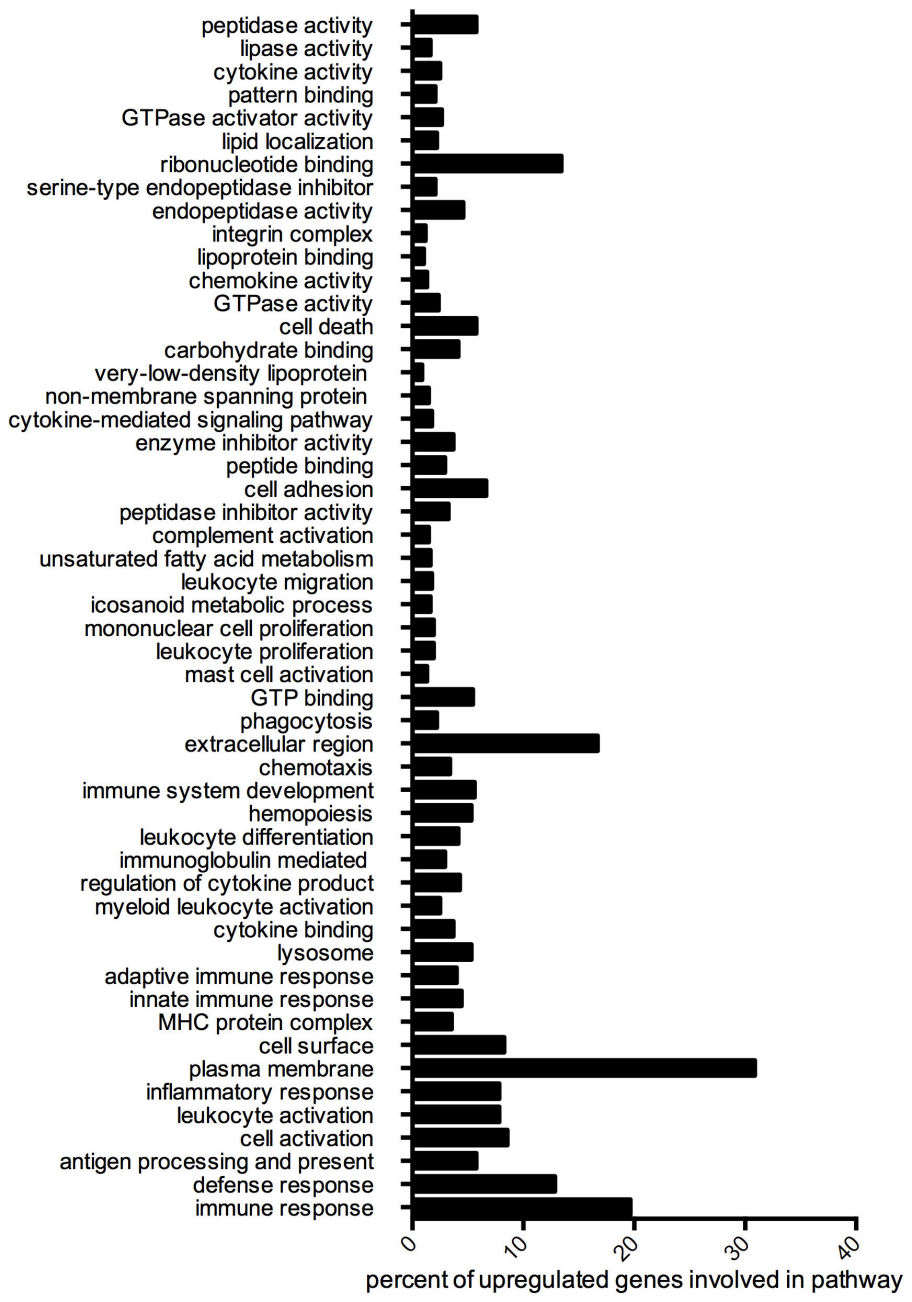
Gene Ontology Analysis. The transcripts for which expression was significantly upregualted (894 transcripts) were analyzed using the functional annotation tool in DAVID and using only the molecular function, cellular component, and biological process terms in the gene ontology database. The most significant and non-redundant categories are represented here. The percentages of the 894 upregulated genes that are involved in each category are represented.

Next, we used the gene ontology (GO) database and the database for annotation, visualization and integrated discovery (DAVID) to characterize the 894 transcripts for which expression was upregulated in infected spinal cord (Table **S2**). Subsets of these genes were found to be involved in antigen presentation and processing, inflammation, defense, and immune responses, chemotaxis and cytokine signaling, complement, and lipid processing, transport, storage, and metabolism (Figure **2** and Table **S3**). A small number of these genes were expressed at undetectable levels in spinal cord from mock-infected mice but at significant levels in spinal cord from MHV-infected mice (65 genes, starred in Table **S2**). These genes encode proteins that perform immunological functions, including surface markers expressed exclusively on myeloid or lymphoid cells (24 genes), cytokines (5 genes), lysosomal components (3 genes), granzyme B, apolipoproteins C-II and C-IV, and a complement component (Cfb). Neither complement components nor active B/T-cells nor their products would be expected to be present in the spinal cord in naïve mice; therefore, these data illuminate the utility of this assay for detecting transcriptional changes reflective of infiltrating immune cells. The 50 most highly differentially expressed transcripts, which are representative of the functional categories in Figure **2**, are listed in Table **2** **.**

**Table 2:** Top 50 Differentially Expressed Genes**.

NCBI Accession***	Gene symbol	Fold Change (log_2_)	P-value
**Antigen presentation**			
NM_010378	H2-Aa	8.091196867	5.24E-38
NM_010545	Cd74	7.559236329	1.23E-37
NM_207105	H2-Ab1	7.164195919	9.97E-33
NM_010382	H2-Eb1	6.078669952	7.39E-26
NM_013532	Lilrb4*	7.153079999	5.53E-20
NM_009857	Cd8a*	7.247316069	5.82E-18
NM_010724	Psmb8	4.256179268	6.92E-15
**Complement**			
NM_021334	Itgax	7.520522753	3.12E-27
NM_009779	C3ar1	5.279252321	2.59E-19
NM_007572	C1qa	4.42367613	3.59E-17
NM_009777	C1qb	4.147839729	1.56E-15
NM_007574	C1qc	4.195323372	1.72E-15
**Associated with lymphocytes (T, B,NK**)			
NM_152839	Igj	7.28755371	1.55E-31
NM_007654	Cd72	6.36344867	5.95E-21
NM_008534	Ly9	6.437381518	5.53E-20
NM_144539	Slamf7	6.979726259	1.10E-19
NM_013706	Cd52	5.404143569	1.87E-19
NM_009850	Cd3g	6.89889651	4.92E-18
NM_009857	Cd8a*	7.247316069	5.82E-18
NM_013489	Cd84	4.961200073	2.36E-17
NM_008147	Gp49a*	8.808431827	6.92E-15
NM_009845	Cd22	5.403189838	6.92E-15
**Cytokines and receptors**			
NM_008599	Cxcl9	8.457703361	7.22E-26
NM_013653	Ccl5	8.138965713	3.68E-23
**Lipid transport and metabolism**			
NM_009853	Cd68*	5.132714539	1.32E-18
NM_009695	Apoc2	5.960434921	1.48E-17
NM_007385	Apoc4	6.306136984	3.17E-16
NM_031195	Msr1*	5.445227457	1.24E-15
**Associated with myeloid cells (macrophages, DCs**)**:**			
NM_053110	Gpnmb*	8.23606446	6.56E-42
NM_009977	Cst7*	7.995478697	5.88E-36
NM_027836	Ms4a7	7.415825374	2.26E-26
NM_010705	Lgals3*	5.361533229	7.20E-22
NM_010821	Mpeg1	4.794347075	2.26E-19
NM_011662	Tyrobp*	4.812736142	4.20E-19
NM_009853	Cd68*	5.132714539	1.32E-18
NM_031254	Trem2*	4.734450912	1.35E-18
NM_145634	Cd300lf*	5.577795412	3.07E-17
NM_009690	Cd5l	7.137739453	3.80E-17
NM_031195	Msr1*	5.445227457	1.24E-15
NM_011426	Siglec1	6.552776952	2.08E-15
NM_008147	Gp49a*	8.808431827	6.92E-15
**Pathogen destruction**			
NM_017372	Lyz2	7.261341918	4.11E-35
NM_013590	Lyz1	7.022574167	1.51E-32
NM_020008	Clec7a	6.380384706	4.54E-26
NM_007807	Cybb	6.264961456	1.38E-25
NM_010705	Lgals3*	5.361533229	7.20E-22
NM_021792	Iigp1	5.022735652	2.36E-17
**Osteoclast function**			
NM_053110	Gpnmb*	8.23606446	6.56E-42
NM_175406	Atp6v0d2	8.109994462	4.53E-25
NM_016873	Wisp2	6.415273428	2.94E-22
NM_013532	Lilrb4*	7.153079999	5.53E-20
NM_011662	Tyrobp*	4.812736142	4.20E-19
NM_031254	Trem2*	4.734450912	1.35E-18
NM_007388	Acp5(TRAP)	6.54687635	4.82E-18
NM_145634	Cd300lf*	5.577795412	3.07E-17
**Other**			
NM_009977	Cst7	7.995478697	5.88E-36
NM_023044	Slc15a3	5.351815911	2.18E-19
NM_019984	Tgm1	6.482855955	3.05E-18
**Unknown function**			
NM_028595	Ms4a6c	5.706251433	2.36E-17
NM_001033767	Gm4951	5.673905156	3.80E-17
NM_001081957	Gm11428	6.094571593	6.44E-16

* These genes are represented in more than one category

** Alternatively spliced isoforms were collapsed into one gene category. The top 50 differentially expressed genes as determined by DESeq are represented. Fold change values are log_2_ transformed.

*** The common gene symbol and the associated NCBI accession number are both listed.

**Figure 3:**
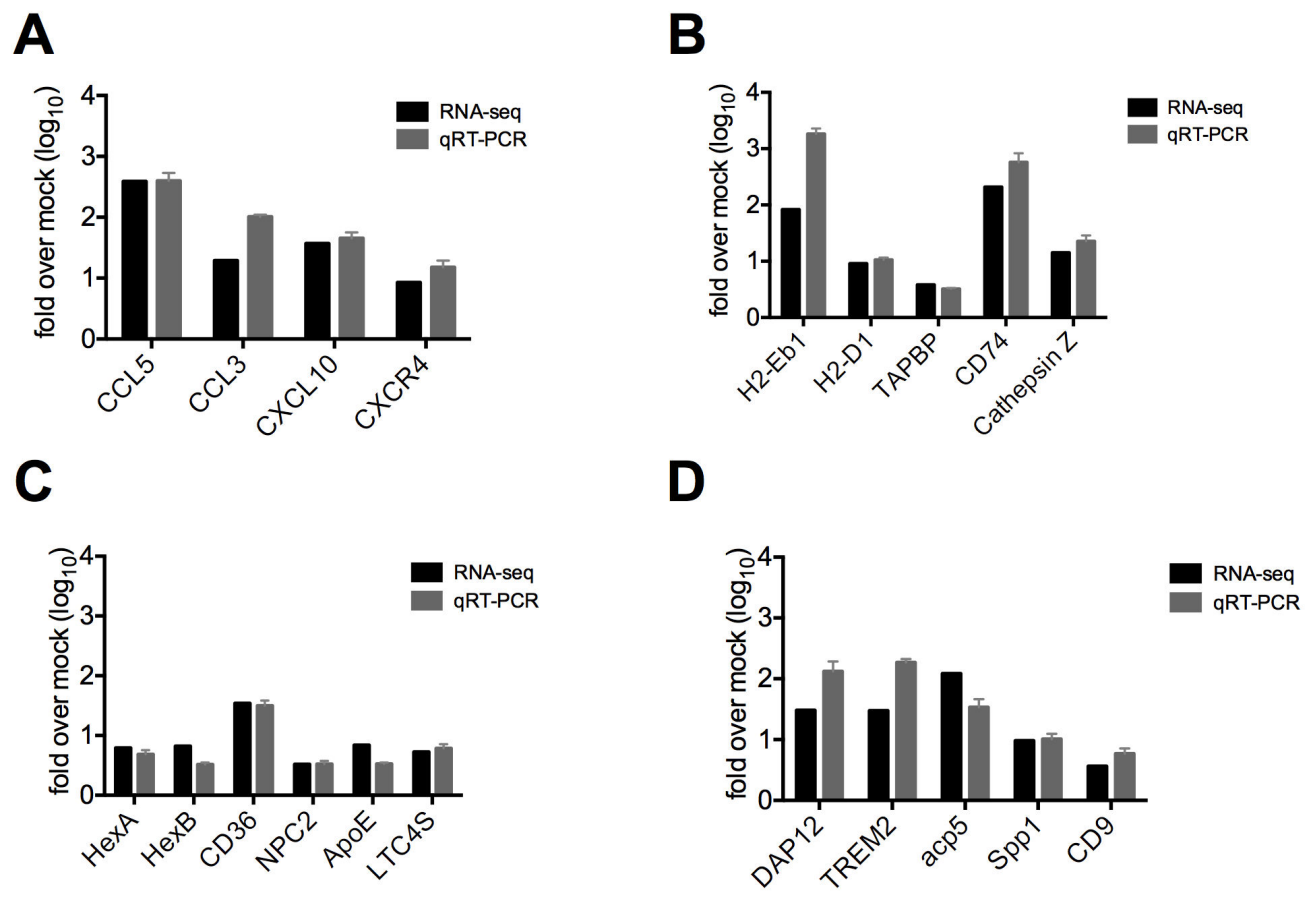
Biological validation of genes of interest. The expression of genes in several categories that were upregulated during MHV induced chronic disease A) Th1 chemokines; B) antigen presentation; C) lipid metabolism and neurodegenerative disease and D) osteoclast differentiation, maturation, and function was quantified by qRT-PCR analysis of spinal cord derived RNA from an independent set of infected mice. Oligonucleotide primers and qRT-PCR were used to detect expression of 4-5 genes in each category. Fold changes of mRNA transcripts in infected mice were determined relative to mock infected and compared to mRNA fold change levels determined in the RNA-seq experiment. All genes analyzed had significantly increased expression in infected mice as compared to mock infected, similar to observations in the RNA-seq analysis. qRT-PCR data plotted are means with SEM of 5 mice.

The RNA-seq results were validated by performing qRT-PCR on several transcripts for which expression was significantly increased in infected tissue and all transcripts assayed exhibited similar fold changes in expression between the two techniques (Figure **S2**). Additionally, total RNA was extracted from the spinal cords of a second set of infected mice and the relative expression of several genes in four categories of genes that were upregulated during demyelination (described below) were validated by qRT-PCR. Thus the RNA-seq data were validated technically by qRT-PCR and biologically by comparison with RNA from an independent set of infected mice (Figure **3**).

### Th1 cytokine and chemokine gene expression is upregulated during MHV-chronic CNS disease

The expression levels of genes encoding several cytokines/chemokines, as well as their receptors, were upregulated in the spinal cords of infected mice harvested 33 dpi (Table **S4**). Twenty-six of these genes encode cytokine or chemokine activities, 29 are involved in cytokine production or regulation, and many others encode proteins involved in cytokine binding or signaling pathways (Figure **2** and Table **S3**). The genes encoding two Th1 chemokines, CXCL9 and CCL5, were among the top fifty most highly upregulated genes (Table **2**). Increased expression levels of four Th1 chemokines were validated by qRT-PCR in biological replicates from mice persistently infected with A59 (Figure **3A**)**.**

### Genes involved in antigen presentation pathways have increased expression during MHV-chronic CNS disease

GO analysis also indicated that expression of 34 genes involved in antigen processing and presentation was upregulated in spinal cord during the persistent phase of MHV infection (Figure **2** and Table **S3**). Twenty of these genes encode major histocompatibility complex alleles, including eight MHC II and 12 MHC I alleles, 14 of which are non-classical (Table **2**, Tables **S3** and **S5**). In addition to MHC alleles, CD74, the gamma chain of the MHC II protein, and CD8α, the MHC I receptor [44], are also highly overrepresented in the spinal cords of persistently infected animals (Table **2**, Figure **3B** and Table **S5**). The increased expression levels of MHC alleles H2-Eb1 and H2-D1, CD74 (a transporter associated with antigen processing binding protein (TAPBP) [45,46]), and cathepsin Z (a lysosomal cysteine protease potentially involved in the degradation of viral antigens [47]), were confirmed by qRT-PCR using the spinal cord of MHV-infected animals in a second experiment (Figure **3B**).

### Genes associated with lipid metabolism and transport have increased expression during MHV chronic CNS disease

We found that MHV infection induced major changes in the transcription of genes involved in many facets of lipid metabolism. In fact, expression levels of 22 genes whose protein products are components of chylomicrons, low-density lipoprotein, or very-low-density lipoprotein particles were increased in the demyelinating spinal cord. Interestingly, expression levels of 12 genes involved in lipid transport, including apolipoproteins E, C1, C2, and C4, were also upregulated (Table **2**, Tables **S3** and **S6**). Eleven other genes for which expression was enhanced in MHV-infected spinal cord encode proteins with lipase or phospholipase activity (Figure **2**, Table **2**, Tables **S3** and **S6**). For example, CD36 [48], CD68 [49], MSR1, and four other genes (Table **S6**) encode macrophage scavenger receptors responsible for phagocytosing macromolecules, cellular debris, and/or oxidized lipid-protein particles.

Eicosanoids, lipid-derived mediators of inflammation that are classified as leukotrienes or prostanoids, are synthesized in response to a variety of stimuli. Several eicosanoid genes in either the prostaglandin or leukotriene pathways showed increased expression in infected CNS tissue (Table **S6**). The expression levels of ten other genes either upstream of or involved in eicosanoid metabolism were upregulated in the MHV infected CNS (Figure **2**, Tables **S3** and **S6**). Additionally, a subset of lipid processing genes that have increased expression in infected tissue include several genes known to be involved in human neurodegenerative diseases (Figure **3C**). Mutations in hexaminidase A, hexaminidase B, Niemann-Pick type C2, and ATP-binding cassette transporter A1 (ABCA1) cause Tay-Sachs [50], Sandhoff disease [51], Niemann-Pick type C2 (NPC2) [52], and Tangier disease [53], respectively.

### Differential expression of oligodendrocyte-associated genes is not associated with MHV- chronic CNS disease

Myelin- and oligodendrocyte-associated genes were not among those expressed at higher levels in MHV-A59 infected versus mock-infected spinal cord (Figure **S3** and Table **S7**). More specifically, genes encoding proteo-lipid protein 1 (PLP1), MBP, myelin oligodendrocyte protein (MOG), myelin-associated protein (MAG), and other structural protein components of the myelin sheath were expressed at similar levels in mock and infected tissue. Similarly, Olig1, 2, and 3 and Sox 8, 9, and 10, which are transcription factors associated with oligodendrocyte differentiation [54-60] (Figure **S3** and Table **S7**), were not more highly expressed in spinal cords of infected mice.

### Increased expression of genes with roles in osteoclast development and function, including TREM2 and DAP12 is associated with MHV chronic CNS disease

Microglia are CNS-resident macrophages and have specialized functions for maintaining homeostasis in their specific environment. Unsurprisingly, 36 genes involved in the differentiation, maturation, and function of myeloid-lineage cells showed increased expression in the 33-dpi MHV-infected spinal cord (Table **2**, Figure **2**, Tables **S2** and **S3**). These genes are involved in scavenging (CD68, MSR1), signaling (Ms4a7, Tyrobp, TREM2, Gp49a, and AIM [61-66]), pathogen destruction (Lgals3 [67]), and monocyte differentiation (Tyrobp, TREM2, Mpeg1, and gpnmb [68-73]). Two of the genes for which expression was most highly induced during MHV persistence were TREM2 (Triggering receptor expressed on myeloid cells 2) and DAP12 (DNAX-activating protein of 12kDa), both of which are involved in myeloid differentiation and function (Table **2**). In humans, congenital natural polymorphisms that cause loss of function mutations in either TREM2 or DAP12 result in Nasu-Hakola disease, which is characterized by late-onset demyelination in the CNS and osteopetrosis [27-30]. Interestingly, several other myeloid-specific genes in addition to TREM2 and DAP12 that exhibited increased expression levels in demyelinating spinal cord are also important for osteoclast differentiation and function (Figure **3D** and Table **S8**). Indeed increased expression of tartrate-resistant acid phosphatase (Acp5), bone sialoprotein (spp1), osteopontin and oncostatin M receptor was associated with chronic MHV induced CNS disease (Table **S4**).

**Figure 4:**
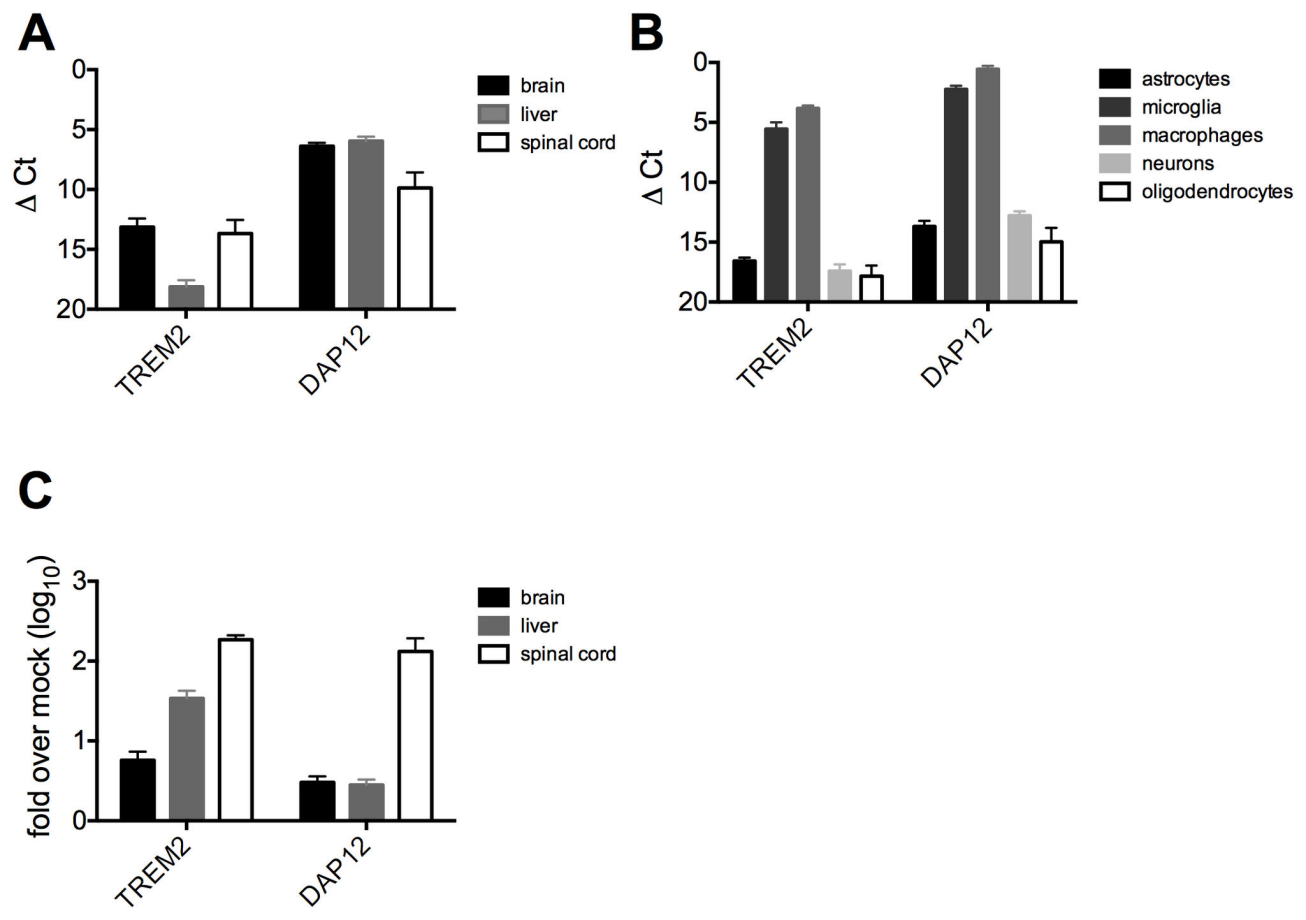
TREM2/DAP12 mRNA expression. A) Expression of TREM2/DAP12 mRNA was determined by qRT-PCR in RNA from spinal cord, brain, and liver of mock infected mice (n=3-5 mice). B) Expression of basal levels of TREM2/DAP12 mRNA was determined by qRT-PCR in RNA from primary cell cultures (oligodendrocytes, n= 2; macrophages, n= 4; neurons, n= 6; astrocytes and microglia, n=5). C) Virus induced levels of TREM2/DAP12 mRNA expression were determined by qRT-PCR in RNA from spinal cord, brain, and liver of infected mice, 33 dpi. Data plotted are means with SEM.

Because natural mutations in TREM2 and DAP12 lead to demyelinating disease in humans and these genes are associated with demyelination in this model, we chose to study the expression of TREM2 and DAP12 mRNA expression levels in the brain, spinal cord, and liver in 33-dpi infected and mock-infected animals (Figure **4A**). In the uninfected mouse, TREM2 is expressed at high levels in the brain and spinal cord, but at minimal levels in the liver, while DAP12 shows higher and more ubiquitous expression in all organs assayed. We also assessed the basal expression levels of TREM2 and DAP12 in CNS cell types cultured *in vitro* (Figure **4B**). DAP12 was expressed in all cell types, with much higher expression in myeloid cells, whereas TREM2 was expressed solely in myeloid cells, with astrocytes, oligodendrocytes, and neurons showing minimal to no detectable expression of this gene (Figure **4B**). In the 33-dpi MHV-infected animals, TREM2 expression was upregulated strongly in the spinal cord and to lesser extents in the brain and liver (Figure **4C**). Infection increased DAP12 expression significantly only in the infected spinal cord and not in the brain or liver (Figure **4C**).

## Discussion and Conclusions

Other and we have reported that MHV genome RNA persists in the CNS for the lifetime of the mouse despite the inability to detect infectious virus during MHV chronic disease [2,74,75]. A previous study comparing persisting and non-persisting strains of MHV indicates that virus-specific CD8T-cells persist only when viral RNA is present and that viral persistence also correlates with demyelination [76]. Therefore, although infectious virus does not appear to persist in the CNS of MHV-infected mice, the viral products that do persist may be the viral mediators of the demyelinating pathology. Here, we report that even by using an extremely sensitive *in vivo* bioassay, persistently infected CNS tissue is unable to transfer infectious virus to naïve hosts. We furthermore demonstrated that viral mRNA as well as genomic RNA persists suggesting viral transcription is occurring and perhaps mRNA is translated into protein that is responsible for maintenance of T cells during persistence as well as the demyelinating pathology. The nature of persistent MHV mRNA is not yet known and requires additional studies, now underway, to characterize the viral sequences, including their mutation rate(s), the presence of six subgenomic mRNAs, leader-junction diversity in subgenomic species, and relative abundance of negative-sense RNA species.

Many different biological activities are hypothesized to lead to demyelination and neurodegeneration in the CNS. Activated lymphocytes and inflammation-mediated responses may directly or indirectly induce death in oligodendrocytes, thus interfering with maintenance of the myelin sheath. The role of oligodendrocytes, the cells that specialize in myelination, in supporting MHV persistence and demyelinating disease has been disputed. Previous studies have shown that MHV persists selectively in oligodendrocytes [11,13,14,77-82]. An early study reported that MHV antigens were present in the oligodendrocytes of demyelinating lesions four weeks post infection [4]. Some studies show that oligodendrocytes are directly damaged during MHV infection but it has also been shown that oligodendrocytes in the MHV infected brain are not apoptotic, and MHV-induced demyelination correlates with inflammation rather than direct damage to oligodendrocytes [14,83,84]. In contrast to MHV infection, immunization against myelin basic protein (MBP) results in direct damage to oligodendrocytes and EAE [85-87] and leads to reduced expression of many oligodendrocyte or myelin-specific genes in the CNS [88,89]. Here we report no changes in expression of oligodendrocyte specific, or myelination-relevant genes during MHV-induced demyelinating disease supporting the hypothesis that oligodendrocytes are not specifically damaged during MHV persistence. It is important to note here that remyelination also occurs during MHV induced chronic disease and that while not maximal until ten weeks post infection, remyelination-associated genes may be upregulated at 33 dpi. However, our data suggests remyelinating processes at this time post infection do not appear to involve increased expression of oligodendrocyte specific genes.

Activated lymphocytes and inflammatory responses can also be damaging to neurons and other cells of the CNS and may contribute to the immune-mediated pathology observed during demyelination. We report here that Th1 chemokines (CCL5, CCL3, CXCL10, CXCR3, CXCR4) have augmented expression during MHV-induced demyelinating disease. CCL5 and CCL3 attract T-cells and other leukocytes to sites of inflammation [90-93], while CXCR4 is a T-cell receptor that promotes chemotaxis to sites of inflammation [94]. CXCL10 (IP-10) and its receptor CXCR3 recruit many immune cells to inflammatory sites and have been previously shown to influence MHV-induced demyelination [17]. Additionally, CXCL10 has been extensively studied in two models of multiple sclerosis, MHV, and experimentally induced autoimmune encephalitis (EAE). Additionally, antibody-mediated blockade of this cytokine has ameliorated disease in both models [17,95]. The combination of increased expression of several canonical Th1 cytokines/chemokines and absent expression of the canonical Th2 cytokines/chemokines [96-101] supports the hypothesis that the host is continuing to mount a cell-mediated immune response to the virus, as previously suggested [102].

Antigen presenting cells activate and recruit lymphocytes to sites of infection and this pathway remains upregulated during MHV-induced demyelinating disease. Several non-classical MHC alleles have augmented expression as well and the roles of these alleles in persistent infection, CNS infection, and demyelination are completely unknown. The MHC II locus is a confirmed multiple sclerosis susceptibility locus in humans [103], and it is possible that certain MHC alleles may also be important for the establishment of MHV-induced demyelination. Although infectious virus does not persist in the CNS, the CD8T-cell response to viral antigen is known to continue in the CNS through this time period [16,76,104,105], and CD8T-cell exhaustion has been proposed to contribute to MHV persistence [106]. Indeed, the sustained activation of lymphocytes, which is associated with sustained antigen presentation, is established not just for MHV-induced demyelination [1,43,107-112], but also for EAE [87,113,114] and multiple sclerosis [115-117], lending evidence to the theory that demyelination is an immunopathological process possibly triggered by diverse biological activities.

The importance of lipid metabolism in general in myelination and demyelination is undisputed. As lipid is a major component of the myelin sheath, changes in lipid metabolism may influence the composition, degradation, or regeneration of myelin in the CNS. Thus, the set of genes related to lipid transport, differentially expressed during MHV-induced chronic disease may be important not only for the formation of the myelin sheath but also for remyelination during MHV infection or in diseases such as MS. Indeed by 33 dpi in the MHV model, while demyelination is the predominant pathology; the remyelination process has likely begun [6]. Thus, increased expression of many lipid processing genes during MHV-induced chronic CNS disease likely represents both processes.

Several genes associated with neurodegenerative disease in humans have highly increased expression during MHV-induced demyelinating disease (HexA, HexB, NPC2, ABCA1, apoE, CD36) [50]. Patients with genetic disorders affecting these genes have greatly reduced amounts of myelination. Furthermore, a variant of apolipoprotein E, apoEε4, is a genetic risk factor for Alzheimer’s disease [118,119], and focal demyelination has been observed near amyloid-beta plaques in both humans and mice [120]. CD36 has been shown to be involved in peripheral nerve remyelination after injury [121]. The expression levels of both CD36 and ABCA1 are also upregulated in demyelinating spinal cord tissue from mice with EAE [88]. In addition, CD36 [122] and apoE [123,124] have recently been associated with the active phase of relapsing-remitting MS.

Additionally, genes involved in the eicosanoid production pathway are also upregulated in the demyelinating CNS. Our data indicate that upregulation of expression of eicosanoid-derived inflammatory mediators may be involved in demyelinating disease, although whether these eicosanoids contribute to or limit the pathology remains undetermined. Regardless, the modulation of eicosanoid-mediated inflammation in demyelinating CNS tissue is under active investigation [125].

Macrophages and microglia promote viral clearance and clear cellular debris; therefore, changes in these cells, as well as in oligodendrocytes, may promote myelin loss and/or alter the host ability to remyelinate during acute infection. Macrophages/microglia have been clearly associated with the pathology of MHV induced demyelinating disease [14,126,127]. A group of myeloid-specific genes that have proven importance for osteoclast function or bone maintenance was also upregulated during MHV-induced demyelination (Table **S4**). Two of the most highly regulated genes were DAP12 and TREM2. DAP12 is a signaling adapter that associates with multiple receptors in different cell types [128] and signals through an ITAM motif [62]. TREM2 signals exclusively through DAP12 and is expressed on myeloid cells in response to unknown ligands [129,130]. Polymorphisms in either of these genes found in individuals with, Nasu-Hakola disease cause demyelination and osteopetrosis, presumably resulting from defects in microglia and osteoclasts respectively [27-30]. DAP-12 deficient mice also exhibit a similar phenotype [131]. DAP12 expression is also associated with NKG2D T-cells in the CNS, which have been demonstrated to contribute to MHV-induced demyelination [132]. Other myeloid-specific genes that were upregulated during MHV induced chronic disease are also important for osteoclast differentiation and function, for example Acp5, Spp1, osteopontin, oncostatinM. Mice deficient in Acp5 exhibit osteopetrosis [133,134]. Bone sialoprotein (Spp1) anchors osteoclasts to bone and is a major component of the bone extracellular matrix [135], while osteopontin has been examined as a modulator of demyelination due to its increased expression in EAE tissue and cerebrospinal fluid from multiple sclerosis patients [136,137]. Lastly, the oncostatin M receptor is also highly expressed in MHV-infected and EAE- and MS-affected tissues [88,138]. Interestingly, infusion of oncostatin M into mice with chemically induced demyelination also regulated the expression of several genes involved in the differentiation of oligodendrocyte precursor cells into mature myelinating cells [139].

Previous studies have confirmed the expression of TREM2 in human microglia, with conflicting results concerning the expression of TREM2 in neurons or oligodendrocytes [140]. Here we show that TREM2 expression is most highly expressed in microglia and macrophages, while DAP12 expression was detected in neurons and astrocytes as well. Interestingly, these genes are more highly induced at 33 dpi in the spinal cord as compared to the brain. This is consistent with studies suggesting that MHV persistence ( [141] and Figure 1) as well as demyelination [142] is to a greater is extent in the spinal cord than in the brain.

Taken together, our findings suggest that osteoclasts and microglia may have several parallel functions. It is known that osteoclasts reabsorb bone (which is created by osteoblasts) using superoxides to destroy the bone matrix and specialized forms of phagocytosis [143,144]. Regulation of osteoclast and osteoblast function is critical for bone homeostasis, and a lack of osteoclast activity caused by mutations in any of several genes leads to osteopetrosis or bone cysts [144,145]. Similarly, microglia, along with inflammatory macrophages, may reabsorb damaged myelin and thus allow oligodendrocytes to remyelinate. Osteoclasts have been reported to present antigen, activate T-cells, and secrete cytokines *in vitro* [146], as microglia have been shown to do in the CNS [147]. Osteoclasts also respond to multiple cytokines that regulate their differentiation and bone-resorptive activity [148], while microglia and inflammatory macrophages are activated by cytokines to scavenge myelin debris [126]. The parallels between osteoclastic and microglial functions in bone remodeling of skeletal tissue and myelin remodeling of the CNS may currently be underappreciated. In support of this model, our findings here indicate a unique role for a subset of bone remodeling proteins in the remyelinating CNS during persistent MHV infection, perhaps by specific expression in activated microglia.

Finally, this study provides the first transcriptome-wide description of gene expression in the spinal cord of mice with MHV-induced demyelination. The data described here provide a basis on which to investigate these pathways in more detail. This includes understanding the pathways contributing to demyelination and/or perhaps remyelination, defining the cell types in which the expression of each of these classes of genes is induced as well as the times post-infection at which each group of genes is upregulated. Further studies will also focus on assessing the influences of translational and post-translational mechanisms that likely constitute another level of the host response to chronic MHV infection.

## Supporting Information

Figure S1 **Expression of RefSeq transcripts.** Expression of each transcript was determined using DESeq in the R statistical background. The current RefSeq database (26,823 transcripts) for *Mus musculus* was divided into four categories: significantly increased expression (log_2_ fold change over mock ≥ and p < 0.05), significantly decreased expression (log_2_ fold change over mock ≤ 1 and p < 0.05), similar expression (-1< log_2_ fold change over mock < 1), and no expression (neither mock nor MHV-A59 infected had any reads to the transcript).
(TIFF)

Figure S2 **RNA-seq technical validation.** The expre≤ssion of nine transcripts was analyzed by RNA-seq and qRT-PCR using the same spinal cord RNAs (experiment 1) and by qRT-PCR of spinal cord RNAs from an independent mouse infection experiment (experiment 2). In each experiment, the samples were obtained from spinal cords of both infected and mock infected mice (3-5 mice), sacrificed at 33dpi. All nine transcripts were significantly induced using both techniques of measurement and in RNA samples from both experiments. T-tests were performed to assess significant induction in the qRT-PCR analysis. Data plotted are means with SEM.
(TIFF)

Figure S3 **Oligodendrocyte-specific gene expression.** As described in the legend to Figure 3, qRT-PCR primers were used to detect the level of expression of oligodendrocyte specific gene transcripts in spinal cords recovered from mock or MHV-A59 infected mice (33dpi). None of the genes had significantly altered expression in infected mice compared to mock. Data shown are means with SEM.
(TIFF)

Table S1 **RNA-seq library statistics.**
(XLSX)

Table S2 **Genes whose expression was increased in the MHV-infected spinal cord.**
(XLSX)

Table S3 **Gene ontology analysis of 894 upregulated genes.**
(XLSX)

Table S4 **Cytokines and cytokine receptors.**
(XLSX)

Table S5 **Antigen Presentation.**
(XLSX)

Table S6 **Lipid and cholesterol transport and metabolism.**
(XLSX)

Table S7 **Oligodendrocyte markers, differentiation and function.**
(XLSX)

Table S8 **Osteoclast and ossification.**
(XLSX)
