# Supplementary figures and images for: Analysis of the Host Transcriptome from Demyelinating Spinal Cord of Murine Coronavirus-Infected Mice
Source: PLoS One. 2013 Sep 18;8(9):e75346. doi: 10.1371/journal.pone.0075346 (PMC3776850; doi:10.1371/journal.pone.0075346)

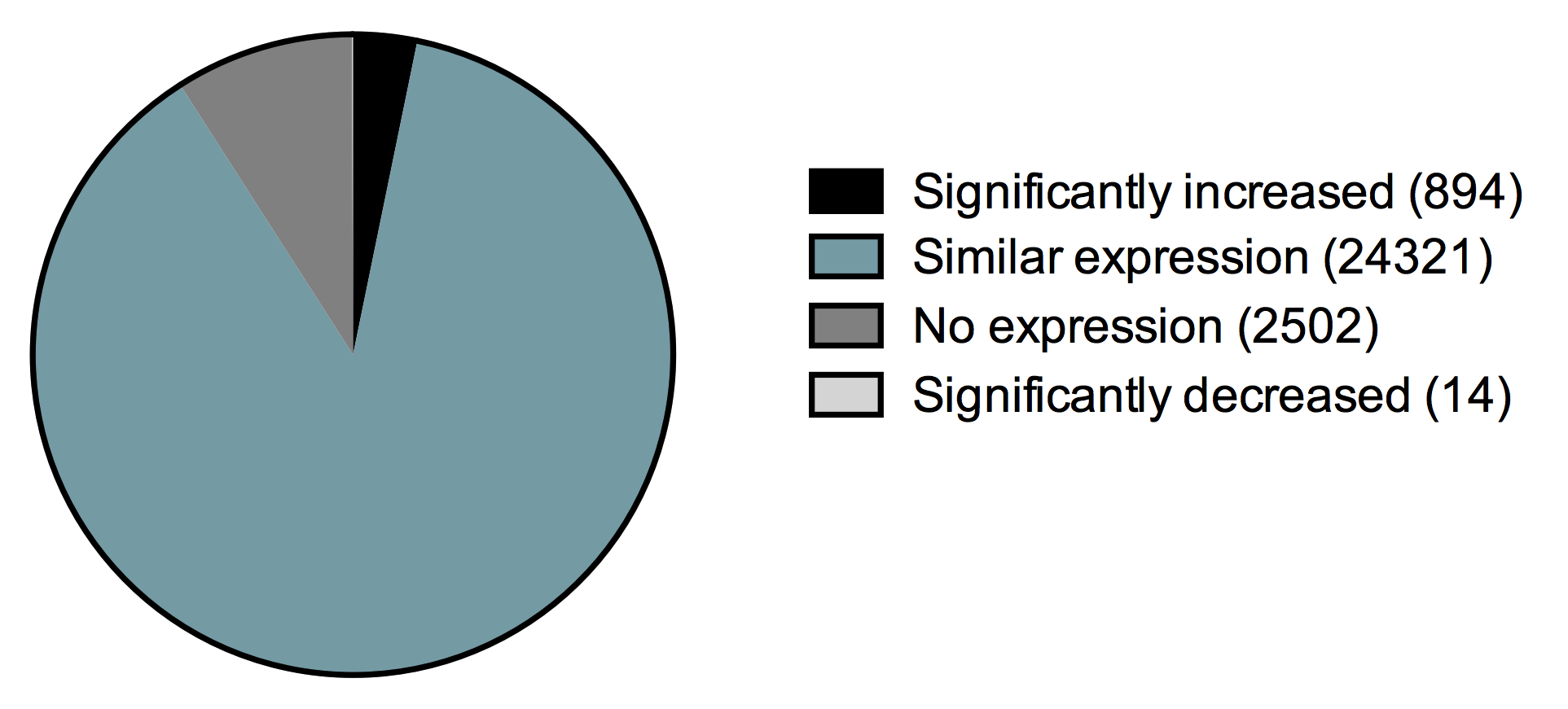

Supplement: Figure S1 — Expression of RefSeq transcripts. Expression of each transcript was determined using DESeq in the R statistical background. The current RefSeq database (26,823 transcripts) for Mus musculus was divided into four categories: significantly increased expression (log2 fold change over mock ≥ and p < 0.05), significantly decreased expression (log2 fold change over mock ≤ 1 and p < 0.05), similar expression (-1< log2 fold change over mock < 1), and no expression (neither mock nor MHV-A59 infected had any reads to the transcript). (TIFF) [file pone.0075346.s001.tiff]

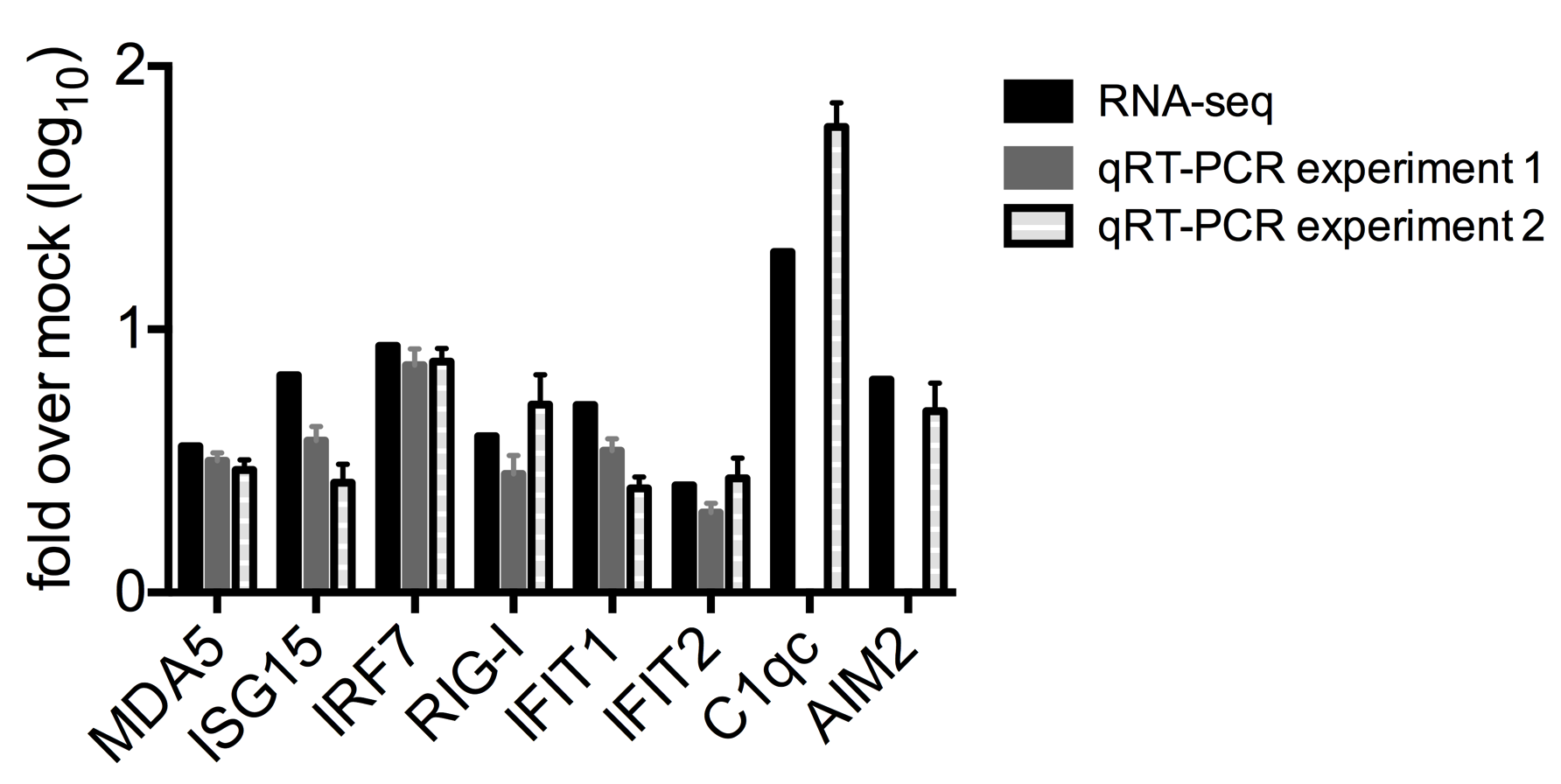

Supplement: Figure S2 — RNA-seq technical validation. The expre≤ssion of nine transcripts was analyzed by RNA-seq and qRT-PCR using the same spinal cord RNAs (experiment 1) and by qRT-PCR of spinal cord RNAs from an independent mouse infection experiment (experiment 2). In each experiment, the samples were obtained from spinal cords of both infected and mock infected mice (3-5 mice), sacrificed at 33dpi. All nine transcripts were significantly induced using both techniques of measurement and in RNA samples from both experiments. T-tests were performed to assess significant induction in the qRT-PCR analysis. Data plotted are means with SEM. (TIFF) [file pone.0075346.s002.tiff]

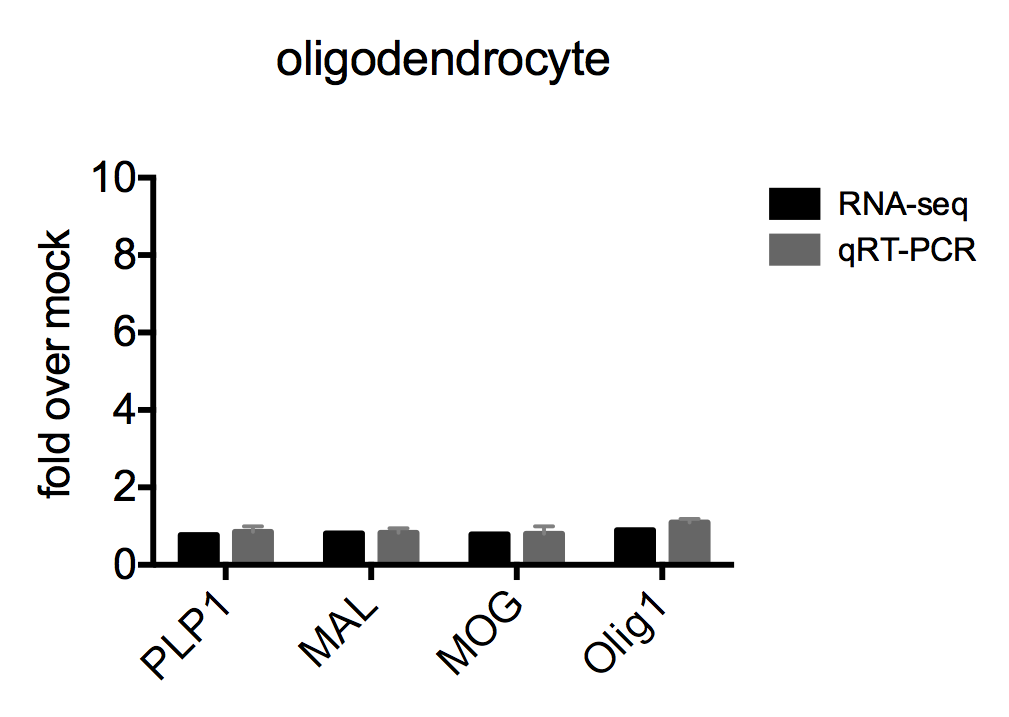

Supplement: Figure S3 — Oligodendrocyte-specific gene expression. As described in the legend to Figure 3, qRT-PCR primers were used to detect the level of expression of oligodendrocyte specific gene transcripts in spinal cords recovered from mock or MHV-A59 infected mice (33dpi). None of the genes had significantly altered expression in infected mice compared to mock. Data shown are means with SEM. (TIFF) [file pone.0075346.s003.tiff]
